# Side Effects and Its Management in Adjuvant Endocrine Therapy for Breast Cancer: A Matter of Communication and Counseling

**DOI:** 10.1177/11782234221145440

**Published:** 2023-01-20

**Authors:** Aina Johnsson, Kerstin Fugl-Meyer, Pal Bordas, Janet Åhman, Anna Von Wachenfeldt

**Affiliations:** 1 Department of Oncology-Pathology, Karolinska Institutet, Stockholm, Sweden; 2 Department of Neurobiology, Care Sciences and Society, Karolinska Institutet, Huddinge, Sweden; 3 Department of Function Area Social Work in Healthcare, Karolinska University Hospital, Stockholm, Sweden; 4 Department of Radiology, Norrbotten Mammography Screening Program, Sunderby Hospital, Luleå, Sweden; 5 Department of Radiation Sciences, Oncology, Umeå University, Umeå, Sweden; 6 Department of Oncology, Södersjukhuset, Stockholm, Sweden; 7 Department of Clinical Science and Education, Södersjukhuset, Karolinska Institutet, Stockholm, Sweden

**Keywords:** Breast cancer, anti-hormonal side effects, sexuality, professional support, focus groups

## Abstract

**Objective:**

Women with a newly diagnosed hormone receptor-positive breast cancer are offered adjuvant endocrine therapy (AET). Although the treatment reduces the risk of relapse and death not all women are adherent to it. Many factors, including the therapy’s menopausal side effects, can adversely affect adherence to the treatment. This study explores the extent to which women treated with AET perceived that health care providers addressed their side effects.

**Methods:**

Ten focus groups were set up, containing between four to nine women. In total, 58 women participated in the study—45 from the Stockholm metropolitan region and 13 from the scarcely populated Norrbotten region. The interviews were analyzed using qualitative content analysis with an inductive approach.

**Results:**

The women were usually satisfied with the care they received from the health care providers. However, their experiences were more complex when it came to their satisfaction with the care in terms of the menopausal side effects of therapy, sexuality in particular. The participants reported that their healthcare providers rarely asked about sex life-related side effects of the treatment.

**Conclusions:**

Health care providers need to communicate and consult about issues related to their patients’ sex lives following their breast cancer diagnosis and during their treatment.

## Introduction

As the female sex hormone estrogen influences the occurrence of breast cancer (BC), a large proportion of the tumors (80%) have the potential for hormone-dependent growth.^1^ This fact is used in modern endocrine BC therapy by either blocking estrogen’s mechanisms of action in the tumor with tamoxifen or reducing the availability of body-specific estrogen in the tissue with aromatase inhibitors (AI) or by castration treatment with gonadotropin-releasing hormone (GnRH). For premenopausal women undergoing adjuvant endocrine (AET) therapy, tamoxifen treatment is often recommended for 5 to 10 years,^2,3^ sometimes with the addition of GnRH analogue for 3 to 5 years.^4^ Tamoxifen may be considered for postmenopausal women, although these women are more commonly recommended AI for 5 or 7 years.^5^ AET has been successful and has greatly contributed to improved survival in hormone-sensitive breast cancer.^2,3,6,7^

However, AET carries the risk of several side effects that can negatively affect a woman’s daily life such as menopausal side effects, sexual dysfunction,^8,9^ and musculoskeletal syndrome.^10^ Tamoxifen and AI produce different side effects. Tamoxifen treatment, for example, can be associated with hot flashes and sweating in up to half of the treated women, side effects less frequently associated with AI. AI therapy, however, can often be associated with vaginal dryness and sexual dysfunction.^11-13^

Demographic, socioeconomic, health-related, and psychosocial factors have been shown to highly impact adherence.^14-20^ It is also well known that if the illness status itself does not produce symptoms but the treatment produces side effects, there is an imminent risk of poor adherence.^21^ This is also the case with AET. In several studies, only half of the women completed AET.^14,15^

Clinical support has been shown to reduce the risk of nonadherence when the patient’s personal support is moderate or low.^20^ The knowledge that social support from health care providers is important for adherence led us to explore women’s experiences of perceived support from their healthcare providers concerning menopausal side effects of AET.

### Purpose

This study explores the extent to which the women treated with AET perceived that the healthcare providers addressed the menopausal side effects they experienced.

## Method

### Study design

This study is a part of a larger project concerning experience of menopausal anti-hormonal side effects which started in January 2013 and involved three hospitals in Stockholm region and one hospital in Norrbotten region.^9,22-25^ This study focuses on the support women with menopausal side effects of AET receive from health care providers and consists of transcribed data from 10 focus group interviews with 58 women subjected to qualitative content analysis.^26^

The research team consisted of seven representatives from several professions: physicians, sexologists, medical social workers, and nurses. This multidisciplinary approach should enhance the credibility of the analysis. The interview guide was based on the preliminary findings in a medical record and questionnaire cohort study,^22^ a literature review, and the clinical experience within the research group. The interview guide covered menopausal disorders, sexual dysfunction, and support from health care providers. Demographic and treatment-related data were obtained from the women both when they agreed to take part in the study and during the focus group interviews.

Women with a hormone receptor-positive BC were invited to participate in focus group interviews. Swedish-speaking residents of metropolitan Stockholm and of rural Norrbotten who had an ongoing AET started within the past 7 years were invited. Exclusion criteria were AET and/or BC treatment prior to the past 7 years. All women offered chemotherapy had completed it at least 1 year before participation in the focus group interviews.

The reason why we chose to conduct the focus group interviews in metropolitan Stockholm and in rural Norrbotten is because these two regions in Sweden have the greatest demographic differences. Rural Norrbotten is the most sparsely populated northernmost district of Sweden, while metropolitan Stockholm, including Sweden’s capital, is the most densely populated area.^27^

Stockholm metropolitan region has several oncology services, but the scarcely populated Norrbotten has only a single oncology service. Both the women from Stockholm and the women from Norrbotten were scheduled to see oncological specialists approximately as often, but Norrbotten has significantly less access to specialized care service and to general practitioners (GP) care.^28^

The participants were recruited via flyers placed in the waiting areas of the oncology clinics in two hospitals in Stockholm and one hospital in Norrbotten. The Breast Cancer Association in both regions/districts also helped with recruitment by sending emails to their members.

### Participants

In Stockholm, 68 women reported an interest in participating. However, four women were no longer interested at the time of the interviews, three women did not meet the inclusion criteria due to previous cancer treatment and 16 were unable to attend the specific focus group interview session they were invited to. In Norrbotten, 13 women participated in the study. During a focus group interview in Norrbotten one woman revealed she had been treatment for BC prior to the past 7 years, which was an exclusion criteria; however, she was included in the study because she had actively participated in her group and influenced the content of the discussion.

### Interview procedure

The focus group interviews were moderated by the first author, a medical social worker with many years of experience talking to women who have undergone breast cancer surgery. The last author, a consultant in oncology, was the assessor during six of the interviews and a specialist nurse in oncology in the remaining ones.

Eight of the focus group interviews were held in a hospital in Stockholm and two interviews were held in a hospital in Norrbotten. In total, 58 women participated in the study—45 from Stockholm and 13 from Norrbotten. In Stockholm, the number of interested women made it possible to invite the participants to sessions according to age groups and whether they had received chemotherapy. This matching was done to increase homogeneity and facilitate the discussion. The interviews in Stockholm were conducted between October 2013 and January 2014 and the interviews in Norrbotten were conducted in October 2013.

The focus group interviews were carried out following the outline given by Krueger,^29^ who suggested that ideally focus group interviews should include a moderator and an assistant moderator. The moderator is responsible for facilitating the discussions, inviting the participants to speak, and encouraging all the members to participate in the discussions.

### Data analysis

All interviews were audio-recorded and transcribed verbatim. The interviews were analyzed using qualitative content analysis with an inductive approach as described by Patton^26^ and Graneheim and Lundman.^30^ After each interview, the moderator and the assistant moderator discussed the intuitive perception of the interview. The discussion was audio-recorded and written down verbatim. A preliminary analysis of the data was done immediately after each focus group interview.

A goal in qualitative researcher is the attainment of saturation. Saturation occurs when adding more participants to the study does not result in additional perspectives or information.^31^ After 10 conducted focus group interviews, the research group found no further perspective on the participants’ experience of the health care and came to the conclusion that saturation was reached and therefore no new focus groups interviews were initiated.

After achieving saturation, a qualitative content analysis with an inductive approach was made described by Patton^26^ and by Graneheim and Lundman.^30^ Qualitative content analysis focuses on the interpretation of texts and an inductive approach involves an unconditional analysis of texts, for example, people’s narratives about their experiences.^32^ To identify differences and similarities in the interviews, the transcripts were analyzed according to Granheim and Lundman’s^30^ recommendations.

The transcribed text from the interviews was analyzed individually by three of the members of the research team. The texts from the discussions between the moderator and the assistant moderators were also read through. The transcribed text was divided into meaning units. These meaning units were condensed and then abstracted into a code describing the contents of the meaning unit. Codes with similar content were merged into themes and sub-themes. The homogeneity and heterogeneity of the content were checked.

All members of the research team had access to the transcribed material and participated in the final analysis.

Each participant was given a group number and an individual number. Quotations from participants from Stockholm were assigned an “S” and those from Norrbotten an “N.” In addition, each quotation showed whether the woman was younger than 50 years (50−) or 50 or older than 50 years (50+).

## Results

Table 1 lists the sociodemographic and clinical factors. The women from Stockholm were younger, had a higher level of education, had more often completed chemotherapy, and had not switched AET to the same extent as the women from Norrbotten. This information was obtained before the focus group interviews began.

**Table 1.** Sociodemographic and clinical characteristics among studied women treated with adjuvant endocrine therapy.

Characteristics	Stockholm region (n = 45)	Norrbotten region (n = 13)
	n	n
Age		
<50 years	9	0
⩾50 years	36 (Mean [range] 57.7 [36-77] SD 9.91)	13 (Mean [range] 65 [52-78] SD 7.77)
Years with therapy		
<2.5 years	18	10
⩾2.5 years	25	3
Missing*	2 (Mean [range] 2.8 [0.2-6.9] SD 1.55)	0 (Mean [range] 2.4 [0.5-5.0] SD 1.57)
University degree		
Yes	35	5
No	9	8
Missing	1	0
Chemotherapy		
Yes	29	3
No	16	10
Prescribed endocrine therapy		
Tamoxifen solely AI solely Switched therapy Forgot the name of prescribed medication	21 17 6 1	4 1 8

Abbreviations: AI, aromatase inhibitors; SD, standard deviation.

* Two women were unsure of the number of months they had used endocrine therapy.

The atmosphere in the focus group interviews was open and supportive. The discussions in all focus groups were very lively. The women confirmed each other’s statements through nodding and nonverbal vocalizations.

The first step in the analysis was to look for statements that addressed menopausal side effects. The reported menopausal side effects were analyzed to get a picture of the participating women’s explicit experiences. The analysis produced two themes: (1) women’s reported menopausal therapy side effects and (2) women’s experiences of receiving information and support from health care providers.

### The women’s reported treatment side effects

Many of the women reported that they had experienced sweating, hot flashes, vaginal dryness, and sexual dysfunction during the treatment period.

The age of the participants impacted how they reported their experience. In groups with younger women, the discussions were livelier and the statements were more detailed. Most of these women expressed that sweating was the problem they found most difficult to handle. Sometimes the side effects were so extensive that it affected their ability to work and hindered normal social activities.

Sleep disturbances due to sweating and hot flashes were a side effect that negatively affected quality of life. The severity of the experienced problem with vaginal dryness differed: some women reported no vaginal dryness, others reported symptoms without experiencing these as problematic and some women reported the symptoms being problematic in that they negatively affected their sex life. Sexual dysfunction was a frequently reported side effect. Many women described how sexual dysfunction negatively affected their overall satisfaction with life. Others sporadically reported problems involving the female genitals, like bladder infection, frequent urination, vaginal discharge, and vaginal bleeding.

Table 2 presents the number of statements on menopausal problems during the focus interviews. These statements were found both when women talked about their problems, when they talked about the relationship with their husband, and when they described the support from health care providers. There may therefore be multiple statements from the same woman under the respective reported side effect. The frequency of reported side effects was significantly more extensive in women from Stockholm than in women from Norrbotten.

**Table 2.** Number of menopausal side effects reported by studied women treated with adjuvant endocrine therapy.

Side-effect	Stockholm region (n = 45)		Norrbotten region (n = 13)	
	Side-effect (n)	No side-effect (n)	Side-effect (n)	No side-effect (n)
Sweating	27	0	5	1
Hot flashes	43	2	6	0
Vaginal dryness	31	8	5	0
Genital sexual pain during sexual activity	4	0	0	0
Decreased sexual desire	39	1	4	2

### Experiences of information and support from health care providers

Overall, women were satisfied with the care they received from their health care professionals. The experiences of care in terms of side effects related to menopausal therapy, especially sexual dysfunction, were more complex.

The reported obstacles to optimal reduction of side effects could be attributed to the health care providers, to the women themselves, or to the interaction between the women and their healthcare providers. Obstacles associated with the health care provider’s mainly concerned lack of time or that the physicians/nurses did not have an approachable manner that is, the women did not feel comfortable raising these issues.

#### Obstacles to optimal reduction of side effects

##### Lack of relevant information

Nearly all the women reported that they had at some point been informed about the side effects, but it was mostly when the medication was prescribed, before the side effects occurred. When the side effects manifested, the women had problems finding health care providers to whom they could express their concerns. That is, when it came to sexual dysfunction, information and communication were lacking.

When discussing advice from their health care providers on how to manage vaginal dryness, some women reported that they received contradictory information from their oncologist and gynecologist concerning the use of medications which included estrogens. Their oncologist’s advice was also perceived as confusing:

It’s something you might want to know about—why you absolutely cannot use medicine containing estrogen. But is there a systemic effect if you just use gel or a pessary (In the same way as treatment with estrogen patches or estrogen tablets); therefore, do they even have a systemic effect at all? Or just a local effect? Sometimes it feels a bit like, well, you do not really know. (S4:1, 50−)

Some of these women had tested herbal medicine containing phytoestrogens, but there were also many who did not dare to use any herbal medicine not recommended by their oncologist:

There are different kinds of herbal remedies, but my doctor has advised against all forms of herbal medicine because the patient has no control over how much phytoestrogen she takes in. (S8:4, 50+)

Moreover, the women desired information about the long-term side effects of disturbed sexual function. Although many women had received information from their health care service provider (eg, physicians or contact nurses), they did not understand what this information really meant when it came to sexuality and sexual function and what they could do to relieve the side effects.

In the absence of information from health care providers, the women sought other sources of information that could provide them guidance for how to deal with the side effects. Here’s how one of the women answers the question of how she gets the information she lacks from healthcare providers:

From the breast cancer association, the Internet and friends and yes, there is a lot of information, I think. Good. (S1:1, 50+)

Many women still did not know where to turn when it comes to sexuality issues:

I would have wished to ask questions or get information about sex actually. I miss that . . . I don’t even know who to turn to. (S2:4, 50+)

##### Sensitivity of the topic

Some women perceived sexuality issues as sensitive and therefore preferred the healthcare provider to raise the theme:

It must be a much more serious problem to make me feel ready to raise such a sensitive topic. If they [healthcare providers] raise it, it’s easier to explain how you feel. (N2:2, 50+)

So I think it would be a little easier if you got a question, if the doctor would ask us, how is it going? (S5:3, 50+)

Some women felt that the healthcare providers behaved in such a way that that it made them embarrassed:

I would have appreciated pure, straight-to-the-point, ordinary, undramatic and professional information . . . Or just to meet someone who knew how to act properly, instead I felt that I was really embarrassed. (S6:1, 50+)

#### Women’s attitude

The obstacles identified by the women included lacking in interest in discussing the issues for varying reasons and a negative attitude toward their doctors’ recommendations.

Some women said that problems related to sexuality were “too private” to talk about with the health care staff. Notably, two women stated that they never talked about issues of sexuality with their healthcare providers. Here are statements from the women:

Sexuality, that word you don’t form on your lips. (S1:1, 50+)

I think the subject [sexuality] is private and do not want my doctor to ask about it. (S7:2, 50+)

Other women were not interested in following the health professionals’ advice, especially when it came to antidepressants:

After all, these are the type of Cipramil [antidepressant] and so on—what are they called? And in a weak dose they would help quite a lot against this, take the edge of off the sweating. And then I would come back if it became unbearable. But I never cared. We have so many other medicines, so you don’t want to take anything more. (N2:4, 50+)

#### Importance of patient support from healthcare provider to endure treatment

Several women stated how important it was to have support from their oncologist. Two of the women express themselves in this way:

There should be a dialogue with the doctor. And like in my case, no, it’s not acceptable that you try to have a conversation about the whole thing and then find yourself feeling really low and had to take the decision by yourself. (S1:5, 50+)

It is clear that normally it is important to complete treatments, you should do this. But you must be able to have a dialogue with your doctor. (S1:4, 50+)

## Discussion

The overall findings of this study were that women treated with AET found health care providers rather silent when it came to issues related to sexual dysfunctions and also the common menopausal side effect of vaginal dryness. This is in concordance with results reported in studies concerning symptoms after AET.^33,34^ Furthermore, health care providers were seldom found to be supportive about sexual problems related to breast cancer treatment. This could be due to time limitation, that is, short consultations and that the issues might not be prioritized. It could be worth mentioning that Swedish physicians and nurses rarely have sexual medicine/sexology in their curriculum and there are few available sexologists to make referrals to. These factors could limit the initiative to start discussions of sexual matters with patients. Furthermore, lack of clinical social support and its consequences, is a topic that has been documented.^20^ Moreover, the women were usually not comfortable raising the questions themselves and sometimes even fearful of being seen as indelicate. For example, some women felt “genital sexual pain” and “decreased sexual desire” were too sensitive to raise.

There are several studies that highlight the need of information and communication regarding AET and the consequences that arise if this communication is lacking.^25,34-37^ The fact that patients want better support has also been highlighted.^34,38^ Awareness of possibilities of existing help for sexual problems would probably benefit communication between the women and their health care providers. A search for knowledge was obvious as women in the focus group interviews requested advice regarding appropriate and feasible treatments. Furthermore, many women treated with AI were uncertain about whether locally applied estrogen would affect their plasma concentration of estrogen and how this might affect their AI treatment. Some had tested herbal medicine containing phytoestrogen, but others did not dare to use any herbal medicine not recommended by their oncologist. If communication and information had been better, much of the uncertainty experienced by the women in this study would probably have been less.

Most of the women wanted their health care providers to raise the issue of vaginal problems and sexual dysfunctions. This finding agrees with several studies showing that women affected by cancer want to talk about issues related to their sex life.^34,39^ If health care providers do not raise these issues, women are at risk of unnecessary suffering.^35^

According to the number of statements during the focus group interviews, the women from the Norrbotten reported on average fewer side effects than those from Stockholm. Which AET is offered depends, among other things, on whether the women are pre- or postmenopausal. Postmenopausal women also have to some extent the opportunity to change endocrine treatment after consultation with their oncologist.^40^ As the different endocrine treatments have different side effect profiles,^11-13^ a change of endocrine treatment can reduce the perceived problems. An explanation for the differences between Stockholm and Norrbotten therefore might be that on group level, the women in Norrbotten were older than those in Stockholm. Another explanation might be that the women from Norrbotten to a greater extent switched their anti-hormonal therapies.

## Strengths and Limitations

A strength is the enthusiasm and openness of the participants. This finding suggests that these women found it useful to share their treatment experiences in a safe space with trustworthy and competent interviewers. Another strength is the multidisciplinary composition of the research team, which enhances the credibility of the method and analysis and created an opportunity to shed light on the research issues from a variety of perspectives. The large number of focus group interviews and the fact that the interviewed women came from geographically different regions also contribute to credibility. Furthermore, the large number of participants made it possible to divide the women into groups by age and whether they had been offered chemotherapy or not. This illustrates the women’s experiences from further perspectives, which additionally increases credibility.

In this study, there are extensive data and data collection extends over time. This can lead to inconsistency. New insights from the focus group interviews may cause a risk that follow-up questions posed by the moderator change over time. Measures were taken to be aware of this and to carefully follow the interview guide. One may argue that the data are somewhat old but to our knowledge, the care of women with adjuvant breast cancer therapy has not changed. One focus group conducted in Norrbotten involved a woman who did not meet the inclusion criteria as she had been treated for breast cancer on an earlier occasion. This maybe a limitation of the study because she thus had an experience that was not shared by other focus group participants. In this study, we have assumed that the number of claims about menopausal side effects reflects the actual occurrence of these. This may be a limitation as there may be other reasons for the difference between the two regions. A limitation of the study is the fact that only Swedish-speaking participants were recruited, limiting the representativeness and therefore the generalizability of the study.

## Conclusions

To influence the likelihood of the women completing AET, effective and repeated communication in a timely matter seems necessary. Health care providers need to communicate the purpose and benefit of AET treatment as well as side effects and strategies for dealing with these.

Furthermore, the health care providers need to be aware that the support they provide is important for adherence to AET.^20^ Information and counseling from health care providers regarding side effects related to sex-life and pharmacological treatment of vaginal dryness were inconsistent, substandard, and contradictory. This left women confused and with little trust in healthcare services.

Clearly, the health care system needs to deal with these issues to ensure these women do not suffer unnecessarily whilst staying on treatment to improve their disease outcome. Gynecologists, oncologists, and other physicians and nurses need to provide women who undergo AET consistent, pertinent, and up-to-date information about pharmacological therapy for vaginal dryness, including genital sexual pain. The same approach is needed when it comes to herbal remedies.

## Declarations

**Ethical Approval and Consent to Participate:** The ethical procedures followed in the study are in accordance with the ethical principles stipulated in the Helsinki Declaration. All participants provided written informed consent and gave written permission for the interviews to be recorded and concent for publication.

**Consent for Publication:** All participants in the focus groups have given their written consent for the study to be published.

**Availability of Data and Materials:** Data is available at Department of Oncology, Södersjukhuset, Stockholm, Sweden.
